# Supplementary material for: Consequences of the COVID-19 Pandemic and Governmental Containment Policies on the Detection and Therapy of Oral Malignant Lesions—A Retrospective, Multicenter Cohort Study from Germany
Source: Cancers (Basel). 2021 Jun 9;13(12):2892. doi: 10.3390/cancers13122892 (PMC8227890; doi:10.3390/cancers13122892)
Supplement: Supplementary file 1 [file cancers-13-02892-s001.zip › cancers-1242185-supplementary.pdf]

Table S1. TNM classification. Distribution of data from the multicenter analysis.

Period	T1	T2	T3	T4	Total
**LD**	**26**	**23**	**12**	**16**	77
PLD	43	34	25	26	128
LD-Ä	50	42	25	33	151
PLD-Ä	77	61	47	88	274

Period	N0	N1	N2a	N2b	N2c	N2	N3a	N3b	N3	Total
LD	45	7	1	7	1	4	0	6	0	71
PLD	83	15	2	3	3	3	0	10	1	120
LD-Ä	101	6	2	8	8	4	1	7	1	138
PLD-Ä	157	32	5	13	9	21	0	25	1	263

Period	M0	M1	Total
LD	57	0	57
PLD	90	9	99
LD-Ä	122	9	131
PLD-Ä	236	10	246

Period	G0	G1	G2	G3	Total
LD	1	19	47	10	77
PLD	1	18	79	25	123
LD-Ä	6	23	87	25	141
PLD-Ä	11	40	149	55	255

Period	R0	R1	Total
LD	58	2	60
PLD	105	6	11
LD-Ä	118	12	130
PLD-Ä	193	22	215

Period	L0	L1	Total
LD	53	7	60
PLD	102	11	113
LD-Ä	121	9	130
PLD-Ä	198	17	215

Period	V0	V1	Total
LD	56	4	60
PLD	109	4	113
LD-Ä	126	2	128
PLD-Ä	208	4	212

Period	Pn0	Pn1	Total
LD	48	12	60

PLD	93	21	114
LD-Ä	113	15	128
PLD-Ä	175	33	208

Table S2. UICC-Classification. Multicenter analysis.

Period	UICC 0	UICC I	UICC II	UICC III	UICC IVA	UICC IVB	UICC IVC	Total
LD	0	20	15	13	19	6	0	73
PLD	1	40	22	22	24	10	7	126
LD-Ä	0	45	32	20	38	7	7	149
PLD-Ä	0	70	39	38	80	34	6	267

Table S3. Distribution of different treatment modalities. Multicenter analysis.

Period	Surgery	Radiation	Radiation & Chemo	Immunotherapy	Palliative Treatment	Total
LD	63	6	3	1	5	78
PLD	118	3	2	4	7	134
LD-Ä	133	0	11	3	13	160
PLD-Ä	225	5	35	0	15	280

Table S4: Distribution of different diagnosis modalities. Multicenter analysis.

Period	Internal Diagnosis	External Diagnosis	Total
LD	59	19	78
PLD	86	49	135
LD-Ä	112	48	160
PLD-Ä	203	77	280

Table S5: Diagnosis code distribution.

**Period**	**C01**	**C02.0**	**C02.1**	**C02.8**	**C03.0**	**C03.1**	**C04.0**	**C04.1**	**C04.8**	**C04.9**	**C05.0**	**C05.8**	**C06.0**	**C10.8**	**C05.1**	**C09.0**	**C09.1**
LD	1	1	15	3	3	12	11	1	4	2	4	3	9	1	7	0	0
PLD	6	0	26	1	4	28	16	8	13	3	5	2	14	1	6	0	1
LD-Ä	2	2	44	6	9	30	13	5	6	0	8	1	14	6	10	3	1
PLD-Ä	13	4	47	10	18	55	21	13	17	5	12	9	34	9	6	2	5

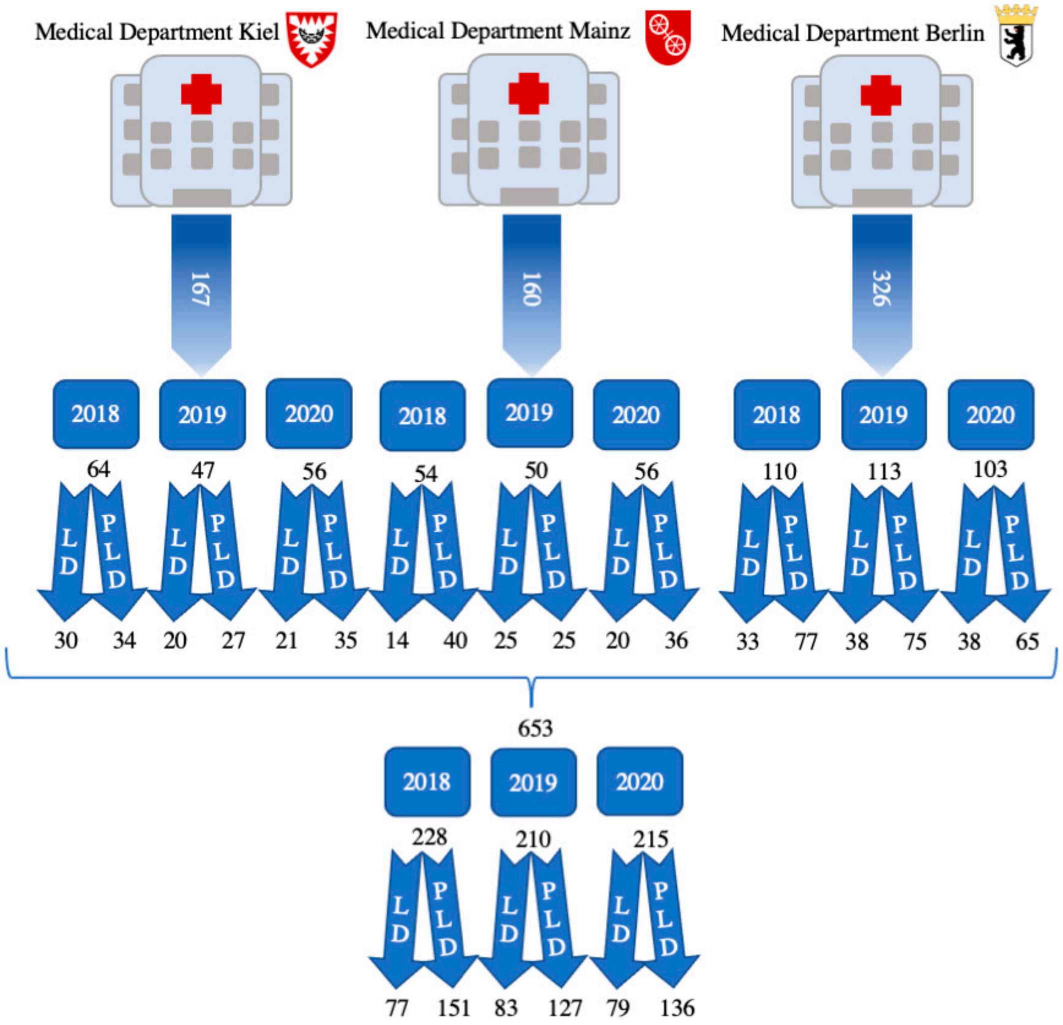

Figure S1. Flowchart. Flow of the data of different medical departments into the analysis.
